# Design of the GOT Doc study: A randomized controlled trial comparing a Guided Self-Help obesity treatment program for childhood obesity in the primary care setting to traditional family-based behavioral weight loss

**DOI:** 10.1016/j.conctc.2021.100771

**Published:** 2021-04-20

**Authors:** Kyung E. Rhee, Lourdes Herrera, David Strong, Anthony M. DeBenedetto, Yuyan Shi, Kerri N. Boutelle

**Affiliations:** a University of California, San Diego, Department of Pediatrics, United States; b Wake Forrest University, Department of Pediatrics, United States; c University of California, San Diego, Department of Family Medicine and Public Health, United States; d University of California, San Diego, Department of Pediatrics, Department of Family Medicine and Public Health, And Department of Psychiatry, United States

**Keywords:** Childhood obesity, Treatment, Primary care, Family-based behavioral therapy, Guided self-help, Community-based intervention, Overweight or obesity, OW/OB, Guided Self-Help, GSH, Family-Based Behavioral Therapy, FBT, Quality of Lifev, QOL, **G**uided self-help **O**besity **T**reatment in the primary care setting, GOT Doc, Early and Periodic Screening, Diagnosis, and Treatment program, EPSDT, U.S. Preventive Services Task Force, USPSTF, Primary care provider, PCP, Electronic health record, EHR

## Abstract

Currently one-third of children in the United States have overweight or obesity (OW/OB). The goal of Healthy People 2020 is to reduce the proportion of children with OW/OB and increase the proportion of primary care visits that include nutrition and weight-related counseling. Unfortunately, many health care providers find it difficult to offer effective weight-related counseling and treatment in the primary care setting. Therefore, new models of care are needed that allow a greater proportion of children with OW/OB and their parents to access care and receive quality weight management treatment. The current paper describes the GOT Doc study which is designed to test the effectiveness of a Guided Self-Help (GSH) model of obesity treatment that can be delivered in the primary care setting compared to a traditional Family-Based Behavioral weight loss treatment (FBT) delivered at an academic center. We will assess the impact of this program on attendance (access to care) and changes in child BMI percentile/z-score. We will also examine the impact of this treatment model on change in child lifestyle behaviors, parent support behaviors, and parent self-efficacy and empowerment to make behavior change. Finally, we will assess the cost-effectiveness of this model on changes in child BMI percentile/z-score. We believe the GSH intervention will be a cost-effective model of obesity management that can be implemented in community practices around the country, thereby increasing access to treatment for a broader proportion of our population and decreasing rates of childhood obesity.

## Highlights

• Effective childhood obesity treatment is not widely available or easily accessible.
• Guided Self-Help (GSH) model of treatment has been developed for pediatric obesity.
• GSH is easier to implement and can be delivered in the primary care setting.
• GSH can increase access to much needed treatment.
• Evaluation of its effectiveness in the primary care setting is needed.

## 1 Introduction

Currently, one in three children in the United States aged 6–19 years have overweight or obesity, with higher rates among Hispanic and African-American children [1]. Because of the many medical and social co-morbidities [[2], [3], [4], [5], [6]] children with overweight/obesity (OW/OB) incur greater medical costs from frequent lab studies [7], sick visits, and utilization of mental health services [8]. While prevention is necessary, widely available and effective treatments are also needed to help those who are already on the path towards greater health care utilization.

The goal of Healthy People 2020 is to decrease the proportion of children with OW/OB and increase the proportion of primary care visits that include nutrition and weight-related counseling [9]. The Early and Periodic Screening, Diagnosis, and Treatment program (EPSDT) for children enrolled in Medicaid also recommends nutrition counseling at each well-child visit [10]. While primary care providers (PCPs) have been identified as key players in the treatment and prevention of obesity by several national groups [[11], [12], [13]] they often report low confidence in their ability to counsel on this topic, as well as a lack of time and resources [14,15]. The U.S. Preventive Services Task Force (USPSTF) has also noted that moderate intensity counseling may not be deliverable by PCPs within the confines of the current well-child visit, and that these children should be referred to intensive counseling or behavioral interventions to assist with weight loss [16]. Thus, if we are to follow national mandates to provide easily accessible and effective obesity-related treatment, other care models need to be developed.

Current gold-standard treatment for obesity involves family-based behavioral therapy (FBT). These programs are intensive, requiring up to 26 h of treatment over 6 months, and are typically located at tertiary care academic centers. However access to these programs can be difficult due to limits on capacity and location [17]. Furthermore, lack of flexibility in scheduling makes it difficult for families to attend weekly group sessions [17]. In one study, only 28% of the families who were offered this treatment enrolled, and only 63% of those families completed the intervention [18].

We developed a Guided Self-Help (GSH) model of obesity management which is shorter in duration, could be delivered in the primary care setting, and has been shown to be equally effective as FBT [19]. GSH provides structured behavioral management for obesity treatment and is designed as a practical tool for health care providers to implement in the primary care office. Although the program is self-directed, GSH programs offer structure to promote treatment compliance and application, as is suggested by the Expert Committee Recommendations for Stage 2 treatment [12]. Frequency and intensity of visits are also realistic for the primary care setting and resembles Medicare coverage for adults.

The goal of this study is to test whether implementation of a GSH model of obesity treatment in the primary care setting can increase access for families to effective obesity treatment and decrease child BMI percentile/z-score. Given the need to increase access and availability of effective obesity treatment in the community, this intervention may provide useful information regarding the structure of such efforts.

## 2 Study objectives

The goal of the GOTDoc (**G**uided self-help **O**besity Treatment in the primary care setting) study is to test the effectiveness of the GSH model of obesity treatment compared to usual care (family-based behavioral weight control treatment (FBT) in a tertiary care setting) in 200 families with children with OW/OB between the ages of 5 and 13 years old. Research staff or health coaches will deliver GSH in the primary care setting and FBT at the UC San Diego Center for Healthy Eating and Activity Research (CHEAR). The intervention and data collection will occur from January 2017 to December 2019. Primary outcomes will include the proportion of treatment sessions families attend (GSH vs. usual care) and change in child BMI percentile/z-score. Secondary aims will include examination of change in child lifestyle behaviors, parent support behaviors, and parent self-efficacy and empowerment to make behavior changes. Cost-effectiveness of GSH on changes in child BMI percentile/z-score is also a secondary outcome. We hypothesize that there will be greater attendance of treatment in the GSH arm, and that among those who attend treatment, there will be similar changes in weight status between groups. However, because more families will attend GSH, we also hypothesize that in the intent to treat analysis GSH will have a greater impact on child BMI percentile/z-score than usual care (FBT). We also hypothesize that GSH will be more cost-effective than FBT.

## 3 Study design

### 3.1 Trial design

GOTDoc is a two-arm, parallel assignment, pragmatic clinical trial, using a randomized control design, comparing GSH delivered in the primary care setting to traditional FBT delivered in an academic center. The intervention will be implemented in the Children's Primary Care Medical Group of San Diego (CPCMG). Two CPCMG sites with 6 providers at each site were identified in San Diego that have similar socio-economic and ethnic/racial demographic characteristics among their patient population. Parent and child will be randomized within each clinic at a one-to-one ratio to receive GSH at the clinic or intensive FBT for weight loss at CHEAR. Randomization will occur at the level of the individual, allowing us to eliminate the effect of the provider on treatment effects. Randomization will also be stratified by child sex to ensure equal distribution between groups. Assessments will occur at baseline, post-treatment (month 6), and 6-month follow-up (month 12). The primary outcomes of this study are attendance and change in BMI percentile/z-score. Secondary outcomes are change in child lifestyle behaviors, parent support behaviors, and parent self-efficacy and empowerment to make behavior change. We will also examine the cost-effectiveness of the GSH model of treatment on changes in child BMI percentile/z-score.

### 3.2 Participants

Participants in this study will include 5–13 years old children with an age and gender adjusted BMI ≥85th percentile, and their parent or guardian (hereafter referred to as parent). Parents may be normal weight, overweight, or obese. The communities from which these samples will be drawn are largely Hispanic (48.9%–58.2%), with a median income ranging from $49,787 to $65,364, 18.3%–10.4% living below the poverty level, and 73.3%–81.9% with a high school degree or higher. The prevalence of childhood obesity in these neighborhoods ranges from 38.9% to 37.9% [20].

### 3.3 Inclusion/exclusion criteria

Inclusion criteria for this study are: 1) a child between the ages of 5 and 13 years old with a BMI ≥85th percentile for age and gender; 2) parent who is responsible for food preparation willing to participate; 3) parent who can read English or Spanish at a minimum of a 5th grade level; 4) family who is willing to commit to attendance at all assessment visits; and 5) family not moving out of the San Diego area within the time frame of the study. Because this is a pragmatic clinical trial, and to allow for examination of heterogeneity of treatment effects and generalizability, limited exclusion criteria will be applied. However, a few criteria will be applied, including: 1) child who is taking medication that may impact weight; 2) child with physical difficulty that limits his/her ability to exercise; and 3) child with a medical condition for which physician supervision of diet and exercise prescription are needed. Children with a diagnosis of a behavioral or psychiatric disorder (based on parent report), but who have been stable on medication for at least 6 months or engaged in therapy, will be allowed to participate in the program as long as they are potentially able to participate in a group setting.

### 3.4 Recruitment and retention

Children with OW/OB and their parent will be identified in the electronic health record (EHR) based on the child's BMI percentile. PCPs will discuss obesity management with the family (to the extent that they would normally do) and then refer them to the study coordinator via the EHR if the family is interested in participating in a weight control program. The study coordinator will be in the office several days of the week to increase recruitment capacity and conduct the screening and consent process. When the study coordinator is not in the office, she will call the family, screen them for eligibility, and obtain additional demographic and medical history information. Families eligible and interested in the study will be invited back to the office to complete the consent process. If more than one child in the family meets study criteria, the study coordinator will use a random numbers table to select the index child for data collection. However, both children will be able to participate in treatment if they are within the study age range. The parent will complete the consent process and form, and any child age 7 years or greater will provide assent to participate and complete an assent form. This study was approved by the Institutional Review Board of the University of California, San Diego.

After the consent process, eligible families will complete assessments. Parents and children will complete online or paper surveys. Families still in the office will have their height and weight measured, otherwise families will return at a later time to obtain a research height and weight. In order to ensure high retention of our sample, we will request personal e-mail addresses and cell phone numbers as well as contact information for two close friends/relatives to enhance our ability to maintain contact with our participants. All families will receive $25 for each of the baseline, 6-month and 12-month assessments as compensation for time and effort. A systematic protocol will be followed to minimize subject attrition at the assessment visits. Participants will be scheduled by telephone, sent e-mail reminders, and called or sent a text reminder the day before the visit. Missed assessment visits will be rescheduled and followed up at least three times. If necessary, transportation to the clinic will be provided. For treatment visits, a similar reminder system will be used.

### 3.5 Assessment and outcomes measures

All assessments will occur at baseline, post-treatment (month 6), and 6-month follow-up (month 12). Both parents and children will participate in these assessments. Assessments will include anthropometry and self-report questionnaires. Parents participating in the study will be asked to complete assessments on their readiness to engage in weight control behaviors [21,22], self-efficacy to lose weight [21,23], and empowerment to access resources for healthy weight [24]. Parents will also report on their behaviors that support child weight loss efforts [25], and potential self-regulatory mediator variables such as goal setting and adherence to GSH recommendations [[26], [27], [28], [29], [30], [31], [32], [33], [34]]. Finally, parents will report on their child's dietary and physical activity behaviors, and provide information on resources utilization for the cost effectiveness analysis. Medical record abstraction will occur at the end of the study to obtain additional information regarding documented anthropometrics and health care utilization. Data collection will be conducted by trained staff and supervised by the principle investigator.

### 3.6 Measures

#### 3.6.1 Measurements (Table 1)

Anthropometry (child and parent). Child and parent height will be measured using a portable Schorr height board (Schorr Inc, Olney, MD) in duplicate. Height will be recorded to the nearest 0.1 cm for both trials, and the average of the 2 values used for analysis. Body weight in kilograms will be measured in duplicate on a Tanita Digital Scale (model WB-110A). Body weight will be recorded to the nearest 0.1 kg and the average of the 2 values used for analysis. Height and weight will be converted to body mass index (BMI = [kg/m2]). Since children are growing, BMI will be translated to BMI for age percentile score using the CDC growth charts [35,36] and to standardized age and gender referenced BMI (BMI Z-scores) [37].

**Table 1:** Schedule of assessments and measures.

	Measure	Baseline	Post-treatment	6-Month Follow-up
Anthropometry	Height/Weight	X	X	X
Demographics	Demographics and Medical History questions	X		
Parent assessments	Parent Importance, Confidence, & Readiness questionnaire [21] Lifestyle Behavior Checklist (Confidence scale) [23] Parent Resource Empowerment Scale [24] Activity Support Scale [25]	X X X X	X X X X	X X X X
Child Lifestyle Behaviors	Dietary History and Eating Behaviors Godin Leisure Time Exercise Questionnaire [41]	X X	X X	X X
Parent support & weight control behaviors	Frequency of self-monitoring behaviors		X	X
	Parent report of adherence to treatment recommendations		X	X
Enrollment/attendance, Treatment Adherence, Acceptability	Enrollment and Attendance (weekly assessment) Self-monitoring in habit books (weekly assessment) Acceptability		X X	X X X
Cost-effectiveness	Family utilization & cost data Administrative data	X X	X X	X X

Demographic Characteristics (baseline only). At the baseline assessment, parents will complete demographic questionnaires that include items on racial/ethnic identity, sex, income, education, marital status, health history, and medication use.

Parent Importance, Confidence, and Readiness for Change Questionnaire: This questionnaire is a self-report measure that assesses motivation for behavioral lifestyle change. It contains three subscales: importance of change, confidence to make changes, and readiness to change. In a weight control study for children age 7–13 years old and their parents, parental confidence predicted premature participant dropout, early treatment response (5-week child weight loss) and child weight loss at post-treatment [21].

The Lifestyle Behavior Checklist (LBC) — Confidence Scale: The LBC lists 25 weight-related problem behaviors in children aged 4–11 years (e.g., eating too much, watching too much television, complaining about doing physical activity) [23]. For the Confidence scale, parents are asked to rate how confident they feel in managing each of these behaviors (even if not currently occurring) on a 10-point scale. The resultant score is a measure of lifestyle-specific parenting self-efficacy. The LBC Confidence scale has very high internal consistency (α = 0.97) and acceptable test-retest stability (r^s^ = 0.66).

Physical activity-, Diet-, and Weight-related Resource Empowerment scale. This 15-item scale was adapted from the Sprietzer Empowerment Scale [38], and is used to assess parent empowerment around accessing resources for child weight, physical activity, and diet behaviors. The scale assessed empowerment in each of these three domains, asking 5 questions around knowledge of resources, ability to access resources, comfort accessing resources, knowledge of the strategies needed to identify new resources, and ability to obtain those resources. A 4-point scale was provided for the response choices (1 = strongly disagree, to 4 = strongly agree). The scale exhibits high internal consistency with internal reliability scores ranging from *α* = 0.93 to 0.97 [24].

Activity Support Scale for Multiple Groups (ACTS-MG): The ACTS-MG is a 12 item scale that was modified from the original Activity Support Scale [39,40] and tested in multiple racial/ethnic groups. The scale consists of three subscales: logistic support, modeling, and use of community resources. Internal consistency coefficients were acceptable for each subscale ranging from *α* = 0.69 - 0.88 [25].

Dietary History and Eating Behaviors: A dietary history and eating behaviors questionnaire was developed to reflect the dietary recommendations that are made in the GSH and FBT interventions. Parents were asked to reflect on their child's eating behaviors in the last week. Sample questions included: frequency of eating breakfast, consuming sugar sweetened beverages, consuming sweet or salty snacks, consuming 4–5 servings of fruits and vegetables per day; frequency of plating your child's dinner; limiting seconds during a meal; use of a measuring device during meal prep; removing high calorie foods from the house; and removing screens from the kitchen. Response choices involving frequency of eating behaviors during the week included: ‘0–7 days’ or ‘never’ to ‘5 or more times per day’. Parents were also asked to report on average daily portion of fruits and vegetables (asked separately) were consumed per day. Five-point likert scales (0 = never to 5 = always) were also used to ascertain how often a specific stimulus control behavior was used during the week.

Godin Leisure-time Exercise Questionnaire: The Godin Leisure-time Exercise Questionnaire [41] assesses the frequency and occurrence of leisure time physical activity. It asks participants to report the number of times during a typical week they participate in mild, moderate and strenuous exercise for more than 15 min. The Godin Leisure-time Exercise Questionnaire correlates with objective indicators of exercise and physical fitness, and has been shown to be a reliable and valid measure of leisure-time exercise [42].

Treatment adherence and acceptability: In both arms, attendance at treatment sessions, adherence to treatment recommendations, and self-monitoring behaviors (percent of days monitored) for child and parent will be collected using daily habit books and food diaries. Questionnaires developed by the research staff will be administered at the end of the study to assess parent and child liking of the program, engagement in specified behaviors, and any concerns or barriers they had during the program. Additional information will be gathered regarding liking of the condition assignment (GSH vs. FBT).

Medical Record Abstraction: At the end of the study period, de-identified information on patients with OW/OB who signed consent but did not complete the assessment or enrollment process will be obtained to determine if there were differences between those who enrolled vs. those who did not. For those enrolled in the study, information will be gathered regarding frequency of patient weight-related visits (to the PCP or a dietitian) and other health care utilization (including referrals, medications, hospitalizations).

Cost-effectiveness measures. Cost effectiveness is of interest in this trial due to the potential to replicate the proposed GSH model in other primary care settings. The primary perspective for this cost effectiveness analysis will be the societal perspective which captures the broadest range of costs that are pertinent [43]. From a societal perspective, the delivery costs for the intervention arm will include reproduction of intervention study materials, distribution of study materials, intervention staff/coaching time for counseling and follow-up, administrative staff time, patient time and expenditures to receive the intervention, and IT support. Research development and research related overhead costs will be excluded from the estimate of the intervention costs.

## 4 Intervention

Parent-child dyads from each site will be randomly assigned to GSH delivered in the primary care setting or FBT delivered in an academic setting. Two large pediatric practices in San Diego County (specifically located in Chula Vista and Escondido, where rates of childhood OW/OB are 38% and 39% respectively) were recruited to participate in this study. Families randomized to GSH will attend 14 sessions (4 consecutive weekly meetings, and then 10 sessions every other week) spanning a 6-month period. Each session will be 20 min in length except for the first session which can last up to an hour, and both parent and child will attend these sessions. During the session, health coaches will answer any questions about healthy dietary and physical activity behaviors, assist parents with problem-solving, and promote accountability.

Families randomized to FBT will attend 16 weekly sessions and then 4 sessions every other week for a 6-month period. Each session lasts 60 min and are conducted in a group format where parents attend one group and children attend another group. Content of the child group mirrors that of the parent group except that information is delivered in a developmentally appropriate format for children. The same information is provided in both GSH and FBT and cover the core nutrition concepts, physical activity behaviors, and basic behavioral strategies that are effective in weight loss. Intervention topics are listed in Table 2. The overall goal of this program is to help children improve their weight status by losing ½ to 1 lb per week [12]. Parents interested in losing weight are also encouraged to do so since parent weight loss is highly correlated with child weight loss [44].

**Table 2:** FBT vs. GSH session topics.

Week #	FBT Session #	FBT Topic	GSH Session #	GSH Topic
1	1	Introduction	1	Introduction
2	2	Dietary Changes	2	Dietary Changes
3	3	Stimulus Control/The Home Environment	3	Stimulus Control/The Home Environment
4	4	Physical Activity	4	Physical Activity
5	5	Problem Solving	–	–
6	6	Positive Parenting/The Rewards System	5	Motivation System and Parenting Skills
7	7	Lifestyle & Sedentary Behaviors	–	–
8	8	Problem Solving around High-Risk Situations	6	Team Building
9	9	Motivation	–	–
10	10	Team Building	7	Problem Solving
11	11	Review	–	–
12	12	Behavior Chains	8	Behavior Chains
13	13	Body Image & Teasing	–	–
14	14	Tricky Hunger & Emotional Eating	9	Emotional Eating
15	15	Shopping on a Budget	–	–
16	16	Meal Planning	10	Shopping on a Budget and Meal Planning
17	–	–	–	–
18	17	High Risk Situations	11	Lifestyle & Sedentary Behaviors
19	–	–	–	–
10	18	Social Support and Sabotage	12	Body Image & Teasing
21	–	–	–	–
22	19	Relapse Prevention	13	Social Support
23	–	–	–	–
24	20	Graduation	14	Relapse Prevention

### 4.1 Obesity management by the PCP prior to randomization

All clinics within the CPCMG network utilize the EPIC electronic health record (EHR) and have a basic obesity management tool for children. This tool includes: 1) alerts to physicians to address and document weight status in all children older than 2 years with OW/OB; 2) prompts to assess risk for obesity-related medical co-morbidities and relevant family history; 3) a tool to facilitate efficient assessment of parent readiness to make behavior changes; 4) a focused menu of options of strategies for weight loss; 5) alerts to obtain appropriate labs, schedule follow-up visits, and make sub-specialty referrals when necessary; and 6) links to handouts and referrals to weight loss treatment options at the tertiary care center. To determine whether parents are ready to engage in obesity treatment, the PCP (prompted by the EHR) will ask parents of all identified children 3 motivation questions: “On a scale of 1–5 with 1 being ‘not important’ and 5 being ‘very important’, how important is it for you to make changes to help your child lose weight at this time?”; “On a scale of 1–5 with 1 being ‘not confident’ and 5 being ‘very confident’, how confident are you that you can help your child lose weight at this time?”; and “On a scale of 1–5 with 1 being ‘not motivated at all’ and 5 being ‘very motivated’, how motivated are you to help your child lose weight at this time?”. These questions are based on the transtheoretical model of behavior change [45], and patients who report higher scores on these questions have reported greater success in weight loss activities [21,46,47]. Parents who report a 3 or higher on any question will be told about the study and asked if their name and contact information can be sent to the study coordinator. If the parent agrees, referrals to the study coordinator will occur via a link in the EHR and/or in person (if she is in the office that day). The study coordinator will contact the family and screen for eligibility. Once families have completed the consent process and baseline assessments, they will be randomized to the intervention (GSH) or usual care (FBT) group. Randomization will be conducted with a random number generator independent of the investigators and physicians. Participants will be informed of both intervention possibilities to ensure their commitment to both. Due to the clear differences in treatment groups, participants will not be blinded to the treatment arms.

### 4.2 Intervention group: Guided Self-Help for pediatric weight loss

The GSH intervention will include 14 sessions over 6 months to mimic the structure of the proposed Medicare funding for obesity treatment for adults (www.cms.gov). These sessions will last 20 min, except for the first session which can last up to an hour, and take place in the clinic. During the first session, the family will receive a parent treatment manual and an age-appropriate child manual that covers several nutrition and physical activity topics as well as behavioral strategies. During the first treatment meeting, manuals are provided to families, and they are instructed to read each chapter prior to the next meeting. The purpose of the manual is to provide key components of gold-standard family-based behavioral therapy (FBT) [[48], [49], [50]] for childhood obesity in a self-help format. Parent manuals emphasize changing the behaviors of the family, and child manuals emphasize changing their own behaviors, in an age-appropriate manner.

In the first session, the health coach will review the structure of future meetings, review how to complete self-monitoring diaries, and set goals with the families. During subsequent sessions, the health coach will assess patient/parent readiness to engage in behavior change, assess barriers and facilitators to behavior change, engage in behavior change and problem solving, and provide feedback and accountability. The goals of the 20-min meetings are to: a) measure the child and parent's weight and reflect with the parent/child dyad on any eating or physical activity changes that may have influenced weight change (either up or down) between visits; b) clarify the content of the manuals; c) use problem-solving to address barriers to implementation of the behavioral strategies outlined in the manual; d) reinforce effort and success; and e) encourage parents and children to try new behavioral techniques. Parents and children will meet together with the health coach during these 6 months.

Dietary recommendations. GSH utilizes the Traffic Light Diet, which categorizes food by energy content into the three colors of the traffic light. Foods are classified into red, yellow and green based on caloric density and grams of sugar per serving; green foods (go) are low in calories and may be consumed in unlimited quantities; yellow foods (caution) have average nutritional value for foods within their food group and are to be eaten with caution; red foods (stop) are energy-dense and should be limited in quantity [[51], [52], [53], [54]]. Dietary recommendations encourage parents/children to count the number of servings consumed for each traffic light color (red, yellow and green). All reductions in energy intake are shaped over time and decreases in consumption of red foods are reinforced. Children will be encouraged to consume 1000–1200 kcals per day while parents will be encouraged to consume 1200–1400 kcals/day. Additional goals include increasing the number of family meals eaten together and increasing vegetable and fruit consumption.

Physical activity recommendations. GSH recommends increasing both lifestyle activity and structured exercise programs [[55], [56], [57]]. Lifestyle activity goals focus on building increased activity into typical, daily activities, such as walking or bicycling to school, and structured exercise goals stimulate routine, planned exercise. Parents and children are also instructed to decrease sedentary behaviors, such as TV watching, video games or computer usage outside of schoolwork. The goal is to engage in 90 min of physical activity on 5 out of 7 days of the week for children and at least 60 min on 5 out of 7 days of the week for parents.

Behavior change recommendations. Key elements of behavior therapy are provided in GSH, including stimulus control, self-monitoring, goal setting and contracting, strategies for managing high-risk situations, relapse prevention skills, and parent management skills training [58]. Self-monitoring is a fundamental aspect of self-regulation and includes observing and recording of eating and exercise behavior, and has been related to weight loss in children and adults [[59], [60], [61], [62], [63]]. In GSH, parents and children will receive “Habit Books,” and are instructed to record their dietary intake and physical activity each day. During each session, the health coach will review habit books and reinforce families for their efforts at any self-monitoring.

In addition to this skill, families will be introduced to stimulus control. Stimulus control aims to reduce environmental cues associated with calorie intake and inactivity, while also increasing environmental cues linked with healthy eating and physical activity. For example, to facilitate a change in dietary intake, families are encouraged to remove high calorie foods from the home, decrease restaurant eating, and to have a variety of fruit and vegetables easily accessible to children. GSH will also focus on setting both immediate and long-term goals. The health coach will encourage the use of proximal, realistic, and measurable goals. Progress towards goals will be evaluated at each meeting.

Parenting skills, as they apply to eating and exercise behavior, are included in the parent manuals only. These topics are derived from parent management training programs [64], and include information on modeling, positive reinforcement, and reward systems. Parent manuals provide suggestions for constructive methods of reviewing habit books with their children and provide examples of how to model healthy eating and physical activity behaviors for children. Motivation or reward systems include a point system in which the child earns points for meeting previously specified behavioral goals. After points are obtained, the child can trade them for rewards that parents and children mutually agree upon.

Towards the end of the intervention, parents will receive information on problem solving, planning ahead, high-risk situations, mastery of potential relapse occurrences, and the differences between lapses and relapses. Both parents and children will discuss how to problem solve and persist through relapses.

### 4.3 Usual care control group: family-based behavioral therapy (FBT) for pediatric weight loss

Families randomized to the usual care group will be referred to the tertiary care FBT weight control program offered at CHEAR. The tertiary care program is based on the gold-standard FBT for pediatric weight loss [[65], [66], [67], [68]] and includes the same nutrition and physical activity education as well as behavioral strategies delivered in GSH. However, families in FBT will attend 16-weekly, and then 4 bi-weekly group sessions over a 6-month period at our site in La Jolla. Group sessions are conducted separately for parents and children and last 60 min each week. Both parents and children will receive weekly age-appropriate manuals and activities to do at home. Families may continue to follow-up with their PCP (time frame to be determined by the PCP) to follow anthropometric changes.

### 4.4 Treatment fidelity

Health coaches for the GSH intervention and parent and child group leaders in FBT are marriage and family therapy trainees or masters level students. The majority of the staff and health coaches were also bilingual and bicultural. All health coaches attended a one-day training with experts in the field of pediatric weight loss (KNB, KER). These trainings reviewed current information regarding nutrition, physical activity, and behavioral strategies for weight loss, and protocols for delivering FBT and GSH. Health coaches were given treatment manuals and protocols in order to maintain treatment fidelity and quality of care. Prior to the start of the intervention, health coaches shadowed a skilled health coach delivering the program to currently enrolled subjects. During the intervention, all sessions will be audiotaped and 10% of tapes will be reviewed during weekly supervision. During the intervention, health coaches have access to both investigators and research assistants via e-mail and phone should any emergent questions arise in the office.

## 5 Data analysis

5.1. This study is a pragmatic randomized control trial using a one-to-one randomization ratio within each clinic, randomizing at the level of the individual. First, we will track the number of sessions each family attended within each group and examine if there is a difference in the proportion of sessions attended between groups using a generalized linear model (binomial family). For our second primary outcome, we will estimate between-group differences (GSH vs FBT) in BMI percentile/z-score (our second primary outcome) over 6- and 12-month assessments using linear mixed effects (LME) analysis adjusting for corresponding baseline BMI percentile/z-score values. BMI z-score is calculated using the formula: [(BMI/M)^L^ −1]/(L x S). (M = median; L = power in the Box-Cox transformation; S = generalized coefficient of variation; values vary based on age in months of the child) All models will adjust for standard patient characteristics (age, race/ethnicity, sex, insurance type) as planned covariates and any differential effects across clinics (i.e., main effect rather than random effect given limited number of clinics).

As a secondary analysis, we will assess the degree to which treatment assignment (GSH vs. FBT) was associated with change in proposed mediators (considered outcomes in these analyses) including child lifestyle behaviors (e.g., eating and physical activity behaviors), parent self-efficacy, parent empowerment, and parent support behaviors (e.g., parent monitoring, adherence to treatment recommendations) using LME models. For mediation modeling, we first will estimate the effect of allocation on a change in proposed mediators (path ‘a’) at 6-months with adjustment for corresponding baseline values. If significant, we will then add baseline and 6-month terms for mediators to the LME model examining their relationship to the primary outcome of BMI percentile/z-score. The indirect effect of group allocation on BMI percentile/z-score through changes in mediators (path ‘b’) will be computed using a product of coefficients method (path ‘a*b’) along with bootstrap confidence intervals. As an adjunct to the primary analyses, we will also explore attendance patterns using a cox proportional hazards regression model to examine predictors of attrition, defined as missing 3 or more consecutive sessions.

5.2. Cost-effectiveness analysis: The cost-effectiveness analysis (CEA) will follow well-established guidelines developed by Drummond et al. [69] and Haddix et al. [70] and include identification of all relevant costs and consequences for each of the interventions and alternatives, accurate measurement in appropriate effectiveness units, sound valuation, and sensitivity analysis to test uncertainties. The final outcome of the CEA in this study is the cost per BMI unit reduced, evaluating the incremental differences between cost and effectiveness in the GSH model and usual care. The precision of the incremental cost-effectiveness ratio (ICER) will be investigated through sensitivity analyses. To account for the non-parametric nature of the data, we will use bootstrap to create confidence intervals for the mean costs and effectiveness for the comparison. ICER will be developed using the nonparametric permutation. A scatter plot of 5000 bootstrapped ICER will be generated by drawing a random sample with replacement [71]. The CEA results will be presented in a cost-effectiveness acceptability curve. The uncertainty of the parameters will be explored in sensitivity analysis.

5.3. Power Analysis: For this trial, sample size estimates took attrition into account. Our first primary hypothesis is that a greater proportion of families in the intervention group will enter into and attend behavioral treatment for obesity. However, there was lack of evidence from previous studies to conduct a power analysis with this outcome. Therefore, power analyses were based on changes in BMI z-score (our second primary outcome) that was supported by observations in the GSH pilot which estimated a decrease in BMI z-score d = |1.71–1.50|/.30 = 0.667. Empirical power estimates were assessed by generating multivariate random samples that were matched to the expected BMI z-score for each condition and variability over time as observed in our pilot study. We expected decreases in BMI z-score of 0.10 among those enrolling in GSH, and a slight increase of 0.05 in BMI z-score in the usual care group. Each of 1000 simulated data sets was analyzed using a LME (random intercepts: patients within clinics) and specific output saved (e.g., standardized regression coefficients, p-values). The percentage of datasets with significant effects (i.e., p < 0.05) for the primary hypothesis comparing GSH vs. usual care provided a simulation-based estimate of power for the primary hypothesis. With a median between-group effect of −0.39 (s d. = 0.13) across 1000 data sets, the planned design would provide greater than 0.82 power for detecting the effect with allowance for up to 20% lost to follow up [72]. We allow up to 20% loss to follow-up to maintain the integrity of the sample for data analysis, and have been able to limit our loss to follow-up in previous trials to 13–18% of the sample. Given these parameters, we estimated that we would need to recruit 200 families into the trial, 100 in GSH and 100 in FBT. With a sample of 200 to support between-group comparisons of BMI percentile/z-score, we estimate we will have power >0.95 to detect a difference in the proportion of sessions attended in a generalized linear model (binomial) with two-sided alpha <0.05 given that we expect >85% attendance in GSH and 60% attendance in FBT. All analyses of primary outcomes will use an intention to treat sample with analyses of all participants who provided both baseline and post-treatment assessments.

5.4. Missing data: The default handling of missing data in mixed models is to make use of all available information from each participant (i.e., no data or cases are deleted) in estimating model parameters [73]. We plan to assess patterns of missing assessments, evaluate any predictors of missing assessments, and evaluate assumptions of methods for applying models to multiply impute data sets. Multiple imputation would be used in an effort to sustain power to compare treatment arms under conditions of data that is missing at random. This approach is both efficient and unbiased, provided that the missing data mechanism is ignorable, the model is correctly specified, and estimated using full likelihood procedures [73]. This approach is advocated as an optimal approach to handling missing data [74] and can help to reduce potential biases in the data.

## 6 Discussion

The GOT Doc study will test the effectiveness of a Guided Self-Help model of pediatric obesity management that is delivered in the primary care setting. This study will allow us to determine whether this model of obesity management can be delivered successfully in this setting while producing similar BMI percentile/z-score changes as FBT that is delivered in a tertiary care academic setting. We will also assess whether the proportion of families attending GSH in the office setting is greater than those who are attending FBT. If so, we may be able to increase access to effective treatment closer to home and intervene earlier in the disease process when it is easier to treat.

During the study, GOTDoc will also examine change in child lifestyle behaviors, parent support behaviors, and parent self-efficacy and empowerment to make behavior changes to determine potential mediators of effect. The cost-effectiveness of this program on changes in child BMI percentile/z-score will also be examined. This information will be important to health systems and insurance companies as we try to develop more efficient treatment models that can effectively address the obesity epidemic in a wide range of settings.

Since obesity often requires chronic management of behaviors in order to help individuals successfully lose weight or maintain a healthy weight status [75,76], the chronic care model [77] of treatment was utilized when designing this study [78,79]. This model works to improve care by linking patients to different components of the health care system and community resources, implement decision support tools and clinical information systems to assist with this care, and create informed, activated patients who are supported to make behavioral changes via goal-setting and problem-solving. At this time, changes in the electronic health record (EHR), like implementing best practice advisories and clinical decision support tools, have been successfully implemented in several studies [[80], [81], [82]] and demonstrated increased frequency of obesity diagnosis and delivery of nutrition and exercise counseling [81]. However, there is little evidence to suggest that these tools have been helpful in actually decreasing BMI. This is not surprising since EHR changes do not necessarily provide patient support for behavior change.

According to the Behavior Change Wheel [83], successful behavior change is facilitated by interventions that promote motivation to make a change and convey strategies to increase patients’ skills, efficacy, and empowerment to make a change. Implementation of a GSH model of obesity treatment may allow health coaches to successfully support self-management behaviors via the delivery of a structured management plan. Since the care manager or health coach in the chronic care model is often tasked to engage the family in brief behavior change counseling and create activated patients who are effective at self-managing their own care, we proposed to adapt this model by training a health coach to provide a Guided Self-Help (GSH) model [19] of obesity treatment. If successful, we may be able to improve the current healthcare delivery model for childhood obesity and increase access to quality, effective, and evidence-based treatment. Ultimately, this model may improve our ability to increase patient/parent engagement in weight management and decrease child BMI.

## Credit author statement

Kyung E. Rhee: Conceptualization, Funding acquisition, Methodology, Supervision, Writing – original draft. Lourdes Herrera: Investigation, Project administration, Data curation, Writing – review & editing. David Strong: Conceptualization, Software, Formal analysis, Writing – review & editing. Anthony M. DeBenedetto: Writing – review & editing. Yuyan Shi: Software, Formal analysis, Writing – review & editing. Kerri N. Boutelle: Conceptualization, Methodology, Writing – review & editing.

## Funding

This work was supported by a grant from the Health Resources and Services Administration, Maternal and Child Health Bureau (R40 MC29452) to KER. They were not involved in any component of the study design, data collection, analysis, or writing of this manuscript.

## Declaration of competing interest

The authors declare that they have no known competing financial interests or personal relationships that could have appeared to influence the work reported in this paper.
